# Examination of the mechanism of Piezo ion channel in 5-HT synthesis in the enterochromaffin cell and its association with gut motility

**DOI:** 10.3389/fendo.2023.1193556

**Published:** 2023-11-02

**Authors:** Zhenya Zhu, Xiaolong Chen, Shuang Chen, Chenmin Hu, Rui Guo, Yuhao Wu, Ziyu Liu, Xiaoli Shu, Mizu Jiang

**Affiliations:** ^1^ Pediatric Endoscopy Center and Gastrointestinal Laboratory, Children’s Hospital, Zhejiang University School of Medicine, National Clinical Research Center for Child Health, National Children’s Regional Medical Center, Hangzhou, China; ^2^ Department of Gastroenterology, Children’s Hospital, Zhejiang University School of Medicine, National Clinical Research Center for Child Health, National Children’s Regional Medical Center, Hangzhou, China; ^3^ National Center, Children’s Hospital, Zhejiang University School of Medicine, National Clinical Research Center for Child Health, National Children’s Regional Medical Center, Hangzhou, China

**Keywords:** piezo, serotonin, p38, tryptophan hydroxylase 1, gut motility

## Abstract

In the gastrointestinal tract, serotonin (5-hydroxytryptamine, 5-HT) is an important monoamine that regulates intestinal dynamics. QGP-1 cells are human-derived enterochromaffin cells that secrete 5-HT and functionally express Piezo ion channels associated with cellular mechanosensation. Piezo ion channels can be blocked by Grammostola spatulata mechanotoxin 4 (GsMTx4), a spider venom peptide that inhibits cationic mechanosensitive channels. The primary aim of this study was to explore the effects of GsMTx4 on 5-HT secretion in QGP-1 cells *in vitro*. We investigated the transcript and protein levels of the Piezo1/2 ion channel, tryptophan hydroxylase 1 (TPH1), and mitogen-activated protein kinase signaling pathways. In addition, we observed that GsMTx4 affected mouse intestinal motility *in vivo*. Furthermore, GsMTx4 blocked the response of QGP-1 cells to ultrasound, a mechanical stimulus.The prolonged presence of GsMTx4 increased the 5-HT levels in the QGP-1 cell culture system, whereas Piezo1/2 expression decreased, and TPH1 expression increased. This effect was accompanied by the increased phosphorylation of the p38 protein. GsMTx4 increased the entire intestinal passage time of carmine without altering intestinal inflammation. Taken together, inhibition of Piezo1/2 can mediate an increase in 5-HT, which is associated with TPH1, a key enzyme for 5-HT synthesis. It is also accompanied by the activation of the p38 signaling pathway. Inhibitors of Piezo1/2 can modulate 5-HT secretion and influence intestinal motility.

## 1 Introduction

Serotonin (5-hydroxytryptamine, 5-HT) plays an essential role in intestinal homeostasis (1, 2), regulates gut secretion and peristalsis, and performs immune and protective functions. It also plays an indispensable role as a mediator in the modulation of the functions of the digestive organs (3) and brain (4), playing an essential role in food intake, digestion, and mood. Enteroendocrine cells are a group of cells that secrete and regulate the functions of the intestine (5). One subgroup of cells that secrete 5-HT, enterochromaffin cells (ECs) (1), has been extensively studied for secretion mechanisms and effects on other cells. ECs are only a minor component of the intestine; however, their functions are pivotal. Previous studies have manipulated ECs in specific ways directly under *in vivo* conditions (6). Furthermore, several cell lines that could be cultured *in vitro* have also been examined (7). ECs cell lines include BON-1, QGP-1, and others (8–10). These cell lines provide a model for studying the secretory functions of the cells. Several mechanisms have been elucidated in the past few years. The main components include chemical compounds, microbiota, signaling pathway molecules, and membrane surface molecules (5, 7, 9–12).

The intestine senses chemical and mechanical stimuli from the outside world and translates them into their effects, accompanied by specific ion channels such as transient receptor potential (TRP) and Piezo1/2 (13, 14). Piezo channels, which are newly discovered mechanosensitive ion channels, influence 5-HT secretion (6). Modulation of 5-HT secretion by manipulating ion channels has garnered significant interest (15). Piezo2-expressing intestinal epithelial cells and enteric neurons directly regulate intestinal motility (16) and feeding (17). Moreover, Piezo1 regulates the secretion of 5-HT (18). These results suggest that Piezo1/2 is a potential target for the regulation of intestinal function. Ultrasound is a mechanical stimulation that can induce activation of neurons and responses in tumor cells (19, 20), making it a technique for applying mechanical stimulation to cells.

Calcium is an essential mineral that regulates cell growth, motility, signal transduction, and other functions (21). Calcium-permeable mechanosensitive ion channels, such as Piezo1/2, allow a large intracellular influx of calcium quickly. Along with calcium influx, many signaling pathways are activated by calcium. The mitogen-activated protein kinase (MAPK) signaling pathway integrates a wealth of external signals translated into the cells (22, 23). The MAPK signaling pathway coordinates the response to inflammation and controls cell growth and differentiation. Thus, MAPK has been used as a target for the treatment of inflammation and tumors.

In this study, we investigated the effect of GsMTx4 (Grammostola spatulata mechanotoxin 4), a spider venom peptide that inhibits cationic mechanosensitive channels, on 5-HT secretion in QGP-1 cells. We also investigated the mechanisms involved, including those related to the 5-HT synthesis enzyme, tryptophan hydroxylase 1 (TPH1), and the calcium signaling-related p38 MAPK signaling pathway. Furthermore, we investigated the *in vivo* effects of GsMTx4 on intestinal dynamics and inflammation.

## 2 Materials and methods

### 2.1 Animal models

C57/BL6 mice (Shanghai SLAC Laboratory Animal Co. Ltd.) were housed at the Zhejiang University Animal Centre, where they were provided adequate water and food. Light was regulated according to time. Special mouse cages were made, where each mouse had its own space to move around independently and was provided with water and food. C57BL/6 mice were randomly divided into two groups, with 5 mice in each group. The experimental group was administered GsMTx4 via oral gavage, while the control group was given PBS. The procedure for administering carmine red via gavage was performed following the experimental protocol (24). The mice were fasted for 12 h. Then the mice were gavaged with 200 μL of 6% carmine (C1022, Sigma) in PBS with or without 1 mg/kg GsMTx4 (HY-P1410, MCE). Following the gavage procedure, the mice were returned to their cages, and their physiological responses were observed to ensure their well-being. The time of the first appearance of red fecal pellets was recorded. After the experiment, fecal pellets were collected and recorded for their quantity and wet weight, allowing the calculation of the number and weight of fecal pellets per unit time for each mouse. After 48 h of continued feeding following the gavage operation, the mice were euthanized using carbon dioxide, and their intestinal tissues were collected and placed in pre-cooled HBSS solution for subsequent experiments. The experiments were conducted in accordance with the ethical guidelines of Zhejiang University. The ethics approval number is ZJM20230025.

### 2.2 Cell culture

QGP-1 cells (JCRB0183, Japanese Collection of Research Bioresources Cell Bank) were cultured in complete RPMI 1640 medium (C11875500, Gibco) +10% FBS (10270-106, Gibco) and Penicillin-Streptomycin (15140-122, Gibco). Cells were passaged at 80% confluence, treated with 0.25% trypsin-EDTA (25200-072, Gibco) for 1-3 minutes, and inoculated at 1000-3000 in 96-well plates or 8 mm PDL (poly-D-lysine)-treated glass, 10^5^ in 6-well plates or 35 mm dishes, and 2 x 10^5^ in 60 mm dishes. For experimental purposes, DMSO-solubilized GsMTx4 was diluted to different concentrations as mentioned earlier (25), and equal amounts of DMSO were added to the control group. In the calcium imaging experiment, the concentration of GsMTx4 used was 10 nM. In the ELISA experiment, the concentration and incubation time of GsMTx4 varied based on the experimental design. However, in the subsequent experiments involving protein immunoblotting and qPCR, the concentration of GsMTx4 was 10 nM, and the incubation time was 8 h.

### 2.3 Calcium imaging

The calcium imaging procedure was performed with reference to the operation protocol used for ultrasound stimulation of liver tumor cells (19). QGP-1 cells were inoculated on confocal glass dishes and used for calcium imaging. A 1 MHz ultrasound (Olympus) probe was used for ultrasound stimulation. The ultrasound generator was provided by Shenzhen Institute of Advanced Technology, Chinese Academy of Sciences. QGP-1 cells were incubated in Fluo-4 AM (F14201, Invitrogen) for 30 minutes, and 488 nm fluorescence was recorded using a confocal microscope (FV3000, Olympus).

### 2.4 MTT assay

The cells were inoculated in 96-well plates and incubated for 48h. MTT reagent (MB4698, Meilunbio) was added, and incubation was continued for four hours before DMSO was added to dissolve the crystalline material formed. Absorbance at 490 nm was recorded using an iMark microplate reader (Bio-Rad).

### 2.5 ELISA

Cells inoculated in 6-well plates were incubated for 48 h after being treated with different drug concentrations at different times. GsMTx4 was dissolved in the cell culture medium to prepare solutions with drug concentrations of 10 nM and 100 nM. In the concentration gradient experiment, cells were grown in medium containing 0 nM, 10 nM, and 100 nM GsMTx4 for 8 h, and then the supernatant was extracted for further analysis. In the time gradient experiment, cells were grown in medium containing 10 nM GsMTx4 for 15 minutes, 30 minutes, 1 h, 2 h, 4 h, and 8 h, respectively, and then the supernatant was extracted for analysis. For the p38 inhibition experiment, 30 μM Adezmapimod and 10 nM GsMTx4 were added to the culture medium. After 8 h of incubation, the supernatant was collected for analysis. The supernatant was extracted and subjected to centrifugation at 5000 rpm, and then the new supernatant was subjected to 5-HT concentration measurements according to the ELISA assay kit (D751013, BBI) operating instructions.

### 2.6 Western blotting

After 48-72h of cell culture in 60 mm dishes, the drug was added, and then the culture was continued for 8 h. Proteins extracted with RIPA (FD009, Fdbio) were added with protease inhibitors (FD1001, Fdbio), phosphatase inhibitors (FD10021, Fdbio), and 5x loading buffer (FD002, Fdbio). Electrophoresis was performed using running buffer powder (M00138, Genscript) and 4-12% gels (M00652, Genscript), followed by membrane transfer using 0.45μm PVDF membranes. Primary antibodies were incubated overnight, and secondary antibodies were incubated for one hour. ECL (FD8020, Fdbio) was then used for chemiluminescence and was recorded using GeneSys (Gene). The following primary antibodies were used: TPH1 (T0678, 1:2000; Merck), Piezo1 (NBP1-78537, 1:2000; Novus), Piezo2 (NBP1-78624, 1:2000; Novus), p38 (ab178867, 1:1500; Abcam), p-p38 (Ab170099, 1:1500; Abcam), ERK (Ab184699, 1:1500; Abcam), p-ERK (ab201015, 1:1500; Abcam), JNK (Ab179461, 1:1500;Abcam), p-JNK (Ab124956, 1:1500; Abcam), and GAPDH (FD0063, 1:5000; FDbio). The secondary antibodies used were mouse-HRP (ab6728, 1:5000; Abcam) and rabbit-HRP (ab6721, 1:5000; Abcam).

### 2.7 RT-qPCR

After 48h of cell culture in 60 mm dishes, total RNA extraction was performed using RNAiso Plus reagent (9108, Takara), followed by reverse transcription using reverse transcriptase to obtain cDNA. Primer sequences were designed using Primer software (**Table 1**), and fluorescent real-time quantitative PCR was performed using a first-strand cDNA synthesis kit (R211, Vazyme) and SYBR Green Mix (Q121, Vazyme).

**Table 1:** Primer base sequences.

Gene	sequence
*ACTB-F*	GAGACCGCGTCCGCC
*ACTB-R*	ATCATCCATGGTGAGCTGGC
*TPH1-F*	CCCTTTGATCCCAAGATTAC
*TPH1-R*	CATTCATGGCACTGGTTATG
*PIEZO1-F*	ATCGCCATCATCTGGTTCCC
*PIEZO1-R*	TGGTGAACAGCGGCTCATAG
*PIEZO2-F*	TGGACACCATTGACGAGCAT
*PIEZO2-R*	CTTCAGTGTAGCAGCTGGAGAT
*PKCA-F*	GTCCACAAGAGGTGCCATGAA
*PKCA-R*	AAGGTGGGGCTTCCGTAAGT
*IP3R-F*	GCGGAGGGATCGACAAATGG
*IP3R-R*	TGGGACATAGCTTAAAGAGGCA
*MAPK-F*	TACACCAACCTCTCGTACATCG
*MAPK-R*	CATGTCTGAAGCGCAGTAAGATT
*AMPK-F*	TTGAAACCTGAAAATGTCCTGCT
*AMPK-R*	GGTGAGCCACAACTTGTTCTT

### 2.8 The immunofluorescence experiment

The slides were treated with PDL in advance, and the cells were then treated with the drug. Paraformaldehyde was added for fixation and Triton X-100 (X100, Sigma) for permeabilization. The reaction was inhibited using 10% goat serum (SL038, Solarbio), followed by overnight incubation with primary antibody: TPH1 (T0678, 1:200; Merck), Piezo1 (NBP1-78537, 1:200; Novus), Piezo2 (NBP1-78624, 1:200; Novus). Slides were then incubated with secondary antibodies for one hour before confocal microscopy imaging (FV3000, Olympus) using FV31S-SW software. The secondary antibodies used were mouse-488 (ab150113, 1:500; Abcam) and rabbit-488 (ab150077, 1:500; Abcam).

### 2.9 HE staining

The intestines of mice were collected after the gavage of the drugs and then fixed with paraformaldehyde. Dewaxing was performed with the following treatments: xylene (10023418, SCRC) twice for 20 min, 100% ethanol (100092683, SCRC) twice for 5 min, 75% ethanol for 5 min, and rinsing with tap water. Sections were stained with hematoxylin solution (G1003, Servicebio) for 3-5 min and rinsed with tap water. The sections were treated with a hematoxylin differentiation solution and rinsed with tap water. Subsequently, the section was treated with hematoxylin Scott Tap Bluing solution and rinsed with tap water. 85% ethanol for 5 min; 95% ethanol for 5 min. The sections were stained with eosin dye for 5 min. Subsequently, dehydration was performed as follows: 100% ethanol for 5 min twice, and xylene for 5 min twice, Finally, neutral gum (10004160, SCRC) was used to seal. Microscopy inspection, image acquisition, and analysis were performed with confocal microscopy imaging (FV3000, Olympus) using FV31S-SW software.

### 2.10 Statistical analysis

All quantitative data included three or more independent experiments, and all data were recorded as the mean ± SEM. Gray scale images were processed using the ImageJ software. Two groups were compared using the STUDENT *t*-test, and multiple groups were compared using the ONE-WAY ANOVA. Images were drawn using GraphPad Prism software. Differences were considered statistically significant at *P*<0.05.

## 3 Results

### 3.1 Mechanically triggered calcium influx in QGP-1 is blocked by GsMTx4

**Figure 1:**
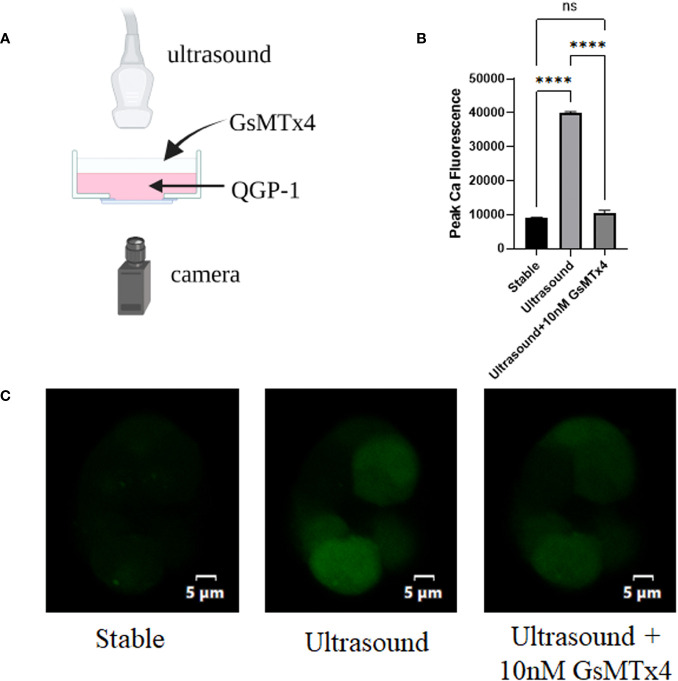
GsMTx4 blocks ultrasound-induced calcium inward flow in QGP-1. The experiment was conducted in QGP-1 cells companied by the immediate addition of 10 nM GsMTx4. **(A)** Ultrasound-based stimulation of cells on confocal glass dishes via the addition of Fluo-4 AM dye to allow calcium signal visualization in the camera (created with biorender.com). **(B)** Calcium signal intensity under ultrasound stimulation with or without the addition of GsMTx4. ****P<0.0001. **(C)** Characteristic images, respectively, at rest (left), under ultrasound stimulation (middle), and after the addition of GsMTx4 (right). ns, not significant.

An ultrasound probe was placed above the inoculated cells on a glass dish. Ultrasound waves were applied to the cells through the water medium **(Figure 1A**). The fluorescence at 488 nm that under conditions involving ultrasonication were approximately three-fold higher than those in the resting state (**Figure 1B**). When 100nM GsMTx4 was added, the fluorescence values did not increase significantly when accompanied with ultrasound stimulation (**Figure 1C**). Calcium activity triggered by mechanical stimulation, such as ultrasound, can be inhibited by GsMTx4.

### 3.2 GsMTx4 increases 5-HT levels and cell viability in the QGP-1 culture system

**Figure 2:**
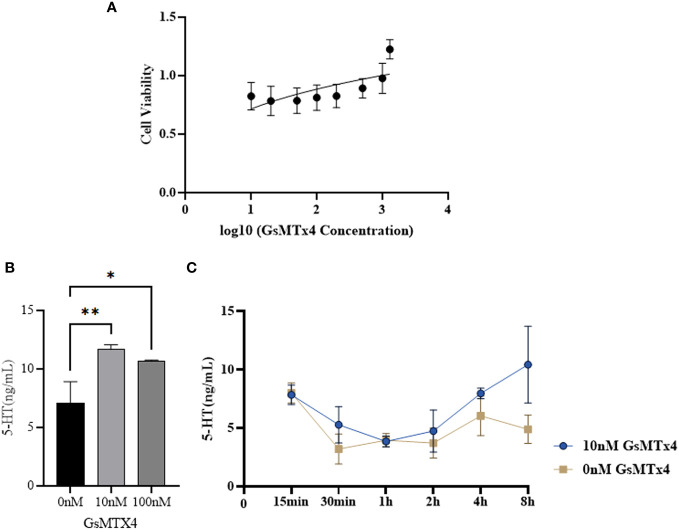
Effect of GsMTx4 on QGP-1 cell viability and 5-HT secretion. **(A)** Cell viability of QGP-1 at different drug concentrations. The ratio of cell viability at different drug concentrations was recorded. **(B)** The concentration of secreted 5-HT in QGP-1 cells after incubation for 8 h with the addition of 10 nM and 100 nM of GsMTx4 (Mean ± SEM, *P<0.05, **P<0.01). **(C)** The concentration of secreted 5-HT in QGP-1 cells after the addition of 10 nM GsMTx4 for different intervals.

The effect of GsMTx4 on the activity of QGP-1 cells was examined using the MTT assay (**Figure 2A**). In the presence of GsMTx4 (10 and 100 nM), the concentration of 5-HT in the drug-treated group was increased by 20-50% with respect to the control group (**Figure 2B**). Time-series experiments showed that at different supernatant in different time phases, the levels of 5-HT in the group with a treatment time longer than 2 h were always significantly higher than those in the control group (**Figure 2C**).

### 3.3 GsMTx4 affected the expression of Piezo1/2 and TPH1

**Figure 3:**
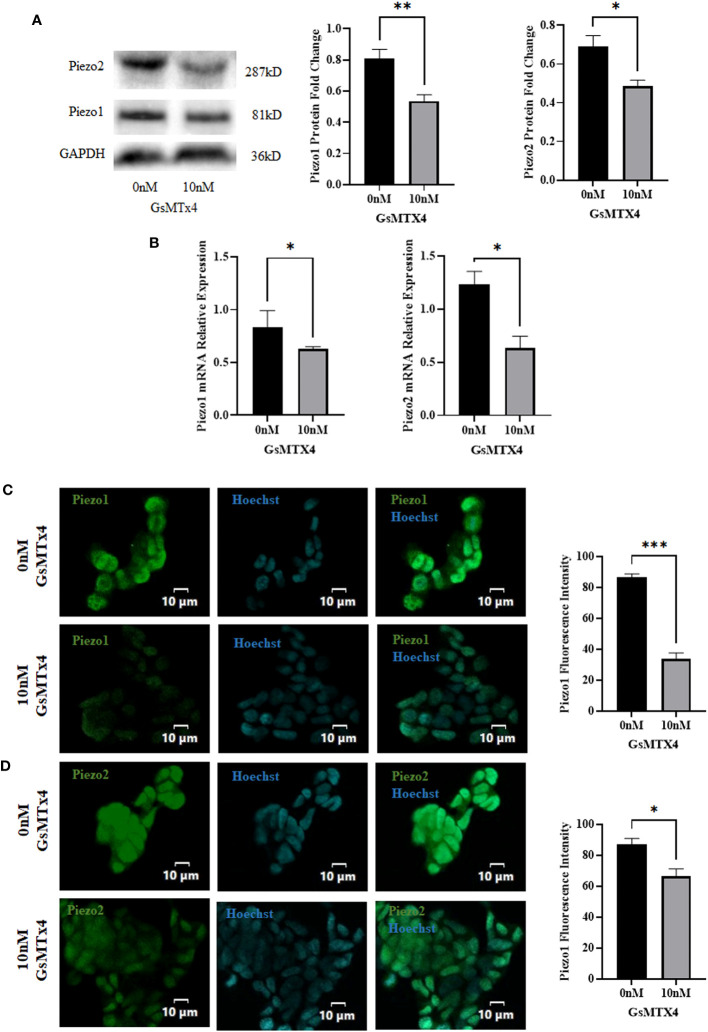
The addition of 10 nM GsMTx4 for 8 h alters the expression of Piezo channels. **(A)** Western blotting experiments of Piezo channels and quantified values (Mean ± SEM, *P<0.05, **P<0.01). **(B)** RT-qPCR experiments of Piezo channels (Mean ± SEM, *P<0.05). **(C)** Immunofluorescence analysis of Piezo1 channels. Nuclei were stained with Hoechst dye.The histogram quantifies the fluorescence intensity (Mean ± SEM, ***P<0.001). **(D)** Immunofluorescence analysis of Piezo2 channels. Nuclei were stained with Hoechst dye.The histogram quantifies the fluorescence intensity(Mean ± SEM, *P<0.05).

We observed the expression of Piezo1/2 after adding GsMTx4 to the culture system. The protein expression of Piezo1 and Piezo2 decreased (**Figure 3A**), along with a reduction in the mRNA expression of *Piezo1* and *Piezo2* (**Figure 3B**). In immunofluorescence experiments, Piezo1/2 was expressed in QGP-1 cells (**Figures 3C, D**), and this expression was widely distributed intracellularly.

**Figure 4:**
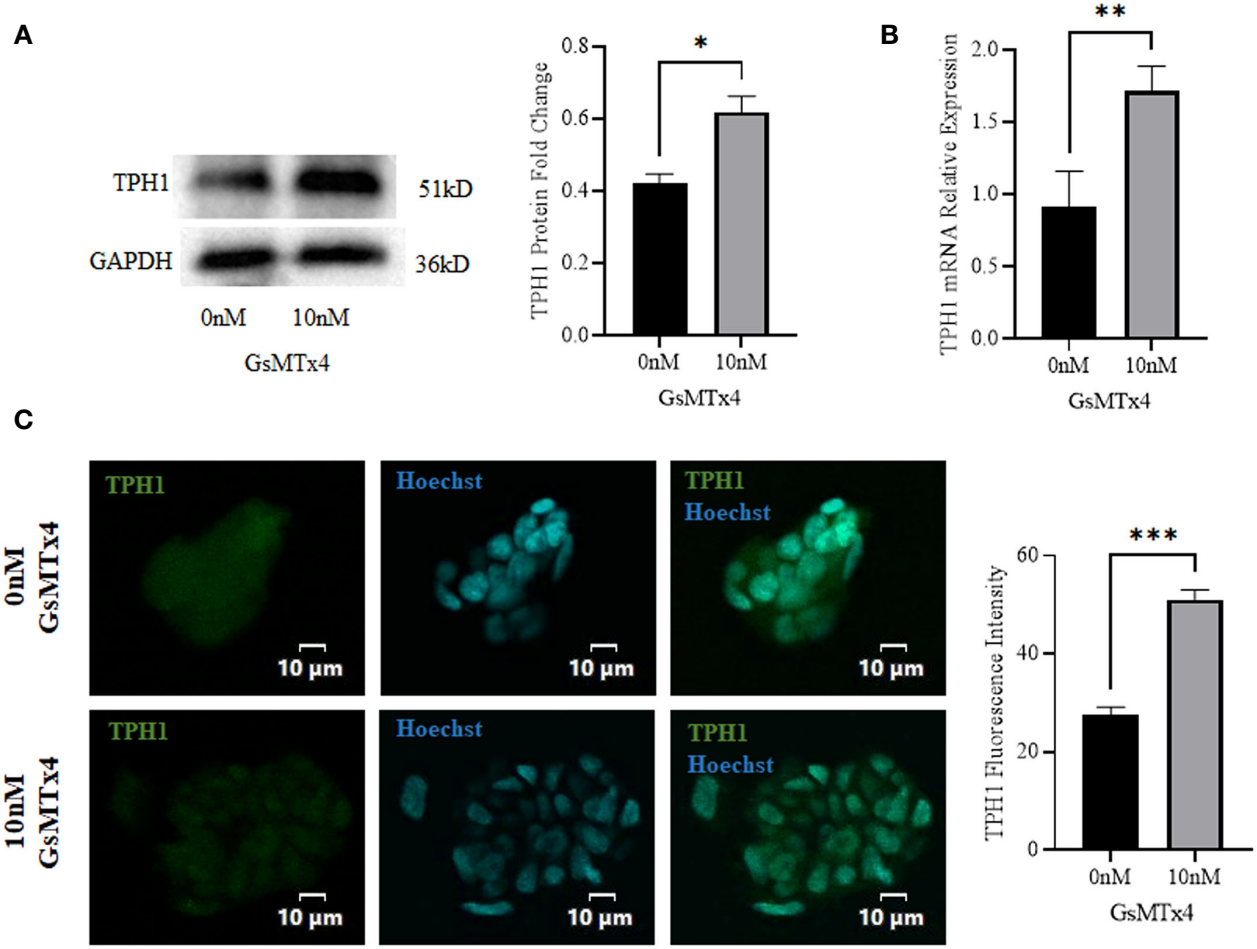
The addition of 10 nM GsMTx4 for 8 h altered the expression of TPH1. **(A)** Western blotting experiments and quantified values of TPH1 (Mean ± SEM, *P<0.05). **(B)** RT-qPCR experiments of TPH1 (Mean ± SEM, **P<0.01). **(C)** Immunofluorescence experiments of TPH1. Nuclei were stained with Hoechst dye.The histogram quantifies the fluorescence intensity (Mean ± SEM, ***P<0.001).

At the protein level, the expression of TPH1 was elevated (**Figure 4A**). The expression of *TPH1* increased significantly at the mRNA levels (**Figure 4B**). In addition, we observed a slight increase in the expression of TPH1 during immunofluorescence analysis (**Figure 4C**).

### 3.4 Increased levels of p38 phosphorylation are accompanied by an excess of 5-HT

**Figure 5:**
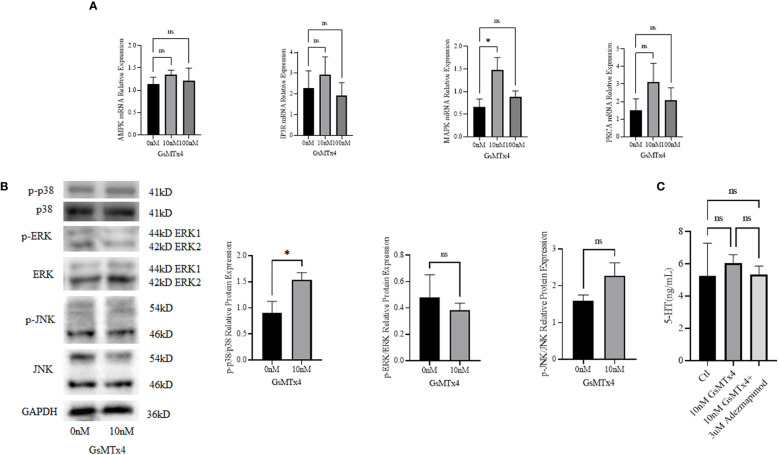
Signaling pathways associated with GsMTx4. **(A)** Differences in mRNA expression levels of calcium-associated signaling pathways with the addition of 10 nM or 100 nM GsMTx4 for 8 h (Mean ± SEM, *P<0.05). **(B)** Protein expression levels of molecules associated with the MAPK signaling pathway with the addition of 10 nM GsMTx4 for 8 h (Mean ± SEM, *P<0.05). **(C)** The concentration of secreted 5-HT in QGP-1 cells after incubation for 8 h with the addition of GsMTx4 or Adezmapimod (Mean ± SEM). Ctl, control; ns, not significant.

We measured the mRNA expression levels of calcium signaling-related *Adenosine 5’-monophosphate (AMP)-activated protein kinase *(*AMPK)*, *MAPK*, *protein kinase C alpha* (*PKCA)*, and *IP3 (inositol 1,4,5-trisphosphate) receptor (IP3R)*, and found that MAPK was upregulated at 100nM (**Figure 5A**). We then investigated the three main marker molecules of the MAPK pathway: p38, extracellular regulated protein kinases (ERK), and c-Jun N-terminal kinase (JNK) (**Figure 5B**) at the protein levels and found that the phosphorylation level of p38 was higher in the GsMTx4 group than in the control group. ERK has two bands, which are the 44 kD ERK1 and the 42 kD ERK2. JNK has two bands, and phosphorylated JNK has two bands, located at 46 kD and 54 kD respectively. JNK produces 10 isoforms through alternative mRNA splicing. At the 46 kD position, there are JNK1α1, JNK1β1, JNK2α1, JNK2β1, and JNK3α1 isoforms. At the 54 kD position, there are JNK1α2, JNK1β2, JNK2α2, JNK2β2, and JNK3β2 isoforms. However, phosphorylation levels of ERK and JNK did not change significantly.

After the addition of GsMTx4, the levels of 5-HT increased, but when GsMTx4 and Adezmapimod were both added, the levels of 5-HT decreased (**Figure 5C**).

### 3.5 Longer intestinal passage times and undifferentiated inflammation were observed *in vivo*

**Figure 6:**
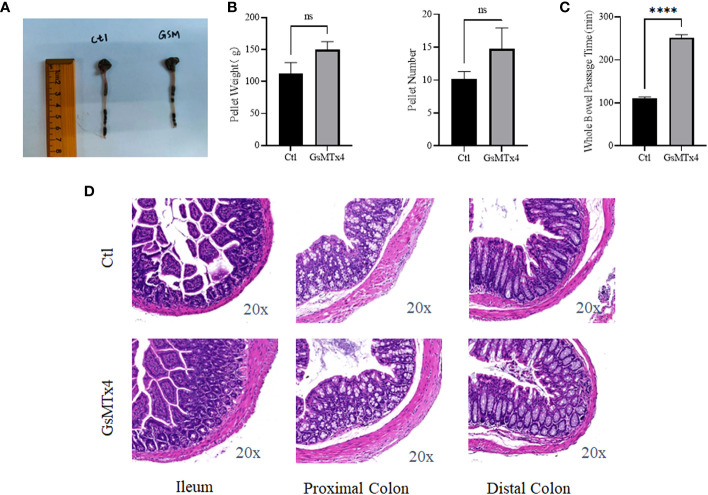
*In vivo* effects of GsMTx4. **(A)** Injection of 1 mg/kg GsMTx4 did not alter the length of the intestine. The drug was administered by oral gavage. **(B)** Stool mass and size (Mean ± SEM). **(C)** Whole intestine passage time (Mean ± SEM, ****P<0.0001). **(D)** HE stained images of the intestine. Ctl, control; ns, not significant.

No significant change in intestinal appearance was observed after the *in vivo* injection of GsMTx4 (**Figure 6A**). Direct administration by gavage increased the whole bowel passage time of GsMTx4 distinctly. The whole bowel passage time in the GsMTx4 administration group was 250 min, while the whole bowel passage time was 110 minutes in control group (**Figure 6C**). The nature of the feces was also examined, and the differences in total mass and water content were not statistically significant (**Figure 6B**). We found no significant inflammation in the small intestine, proximal colon, or distal colon in the control and dosing groups (**Figure 6D**).

## 4 Discussion

**Figure 7:**
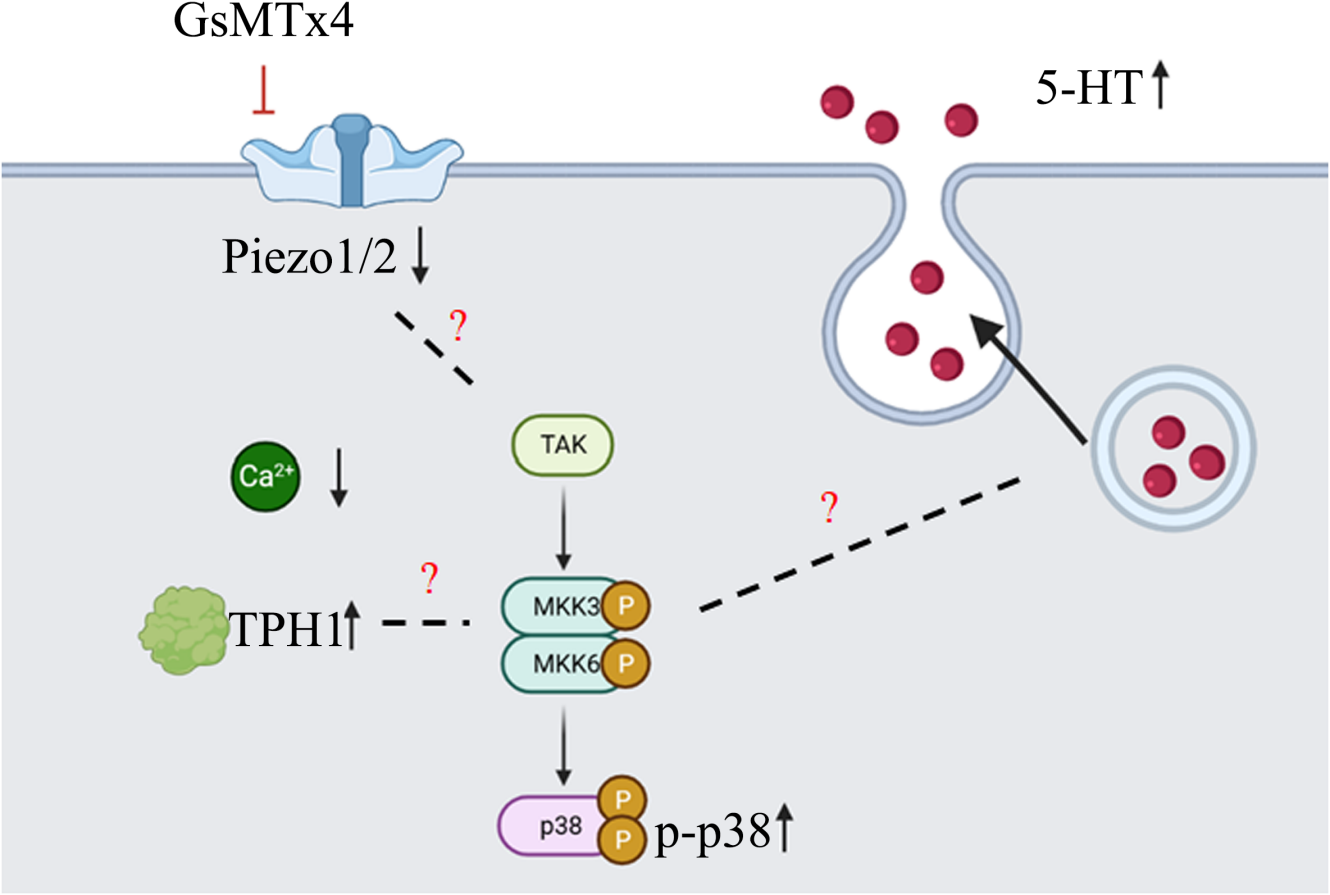
Diagram of the main content of this article (created with biorender.com). GsMTx4 can inhibit the increase of intracellular calcium induced by mechanical stimulation, leading to a decrease in the protein expression of Piezo1 and Piezo2, and an increase in the protein expression of TPH1. During this process, the phosphorylation level of p38 protein increases. At the same time, there is an increase in the secretion of 5-HT. However, there is no sufficient evidence supporting the association between the changes in Piezo1/2 and TPH1 proteins and p38 protein phosphorylation. Additionally, the mechanism underlying the increased 5-HT secretion also requires further experimental research.

Our study found that the addition of GsMTx4 resulted in a small and sustained elevation in 5-HT levels (**Figure 7**). At different drug concentrations (10 nM and 100 nM) and treatment times, an increase in the concentration of 5-HT was observed in the culture system compared to that in the control. This effect correlated with the attenuation of the transient intracellular calcium signal, but the intensity of the long-term calcium signal was not analyzed. Alcaino et al (6) linked the increase in intracellular calcium signaling in enterochromaffin cells to the secretion of 5-HT. Mechanical stimulation led to a transient rise in intracellular calcium signaling, affecting the transport of 5-HT vesicles and triggering 5-HT release by binding to the cell membrane. We also confirmed that QGP-1 cells respond to mechanical stimulation, and this response was blocked by the inhibitor of the Piezo ion channel, GsMTx4. Our experimental results demonstrate that long-term (8 h) inhibition of the Piezo ion channel also leads to an increase in 5-HT secretion, which may be unrelated to the transient inhibition of calcium signaling. Several pathways regulate the 5-HT synthesis and secretion (2–4). The synthesis, secretion, and reuptake of 5-HT are mediated via cascades. Many signaling pathways are associated with TPH1 (26), which directly controls 5-HT synthesis. Various factors can regulate the vesicular transport of 5-HT. The reuptake of 5-HT by serotonin transporters (SERT) can also be modulated (27). The processes of 5-HT synthesis, secretion, and reuptake are related to chemical factors, microbiota, G protein-coupled receptor (GPCR), ion channels, and signaling pathways (28). Our experiments provide an indirect method of producing 5-HT using drugs capable of influencing Piezo1/2, which affects 5-HT secretion. This effect is significant and suitable for obtaining high levels of states with low amplitudes and persistent 5-HT concentrations.

Subsequently, we investigated the mechanisms underlying the elevation in 5-HT levels (**Figure 7**). GsMTx4 is an inhibitor of Piezo1/2 that block channel function (29). We investigated the expression of Piezo1/2 ion channels; the expression of both channels was reduced. Thus, the pharmacological effects of GsMTx4 included not only functional inhibition but also downregulation of Piezo1/2 ion channel expression. Piezo1, an ion channel widely expressed throughout the body, regulates 5-HT levels by sensing RNA (18). In contrast, ECs directly translates mechanical forces into 5-HT secretion via Piezo2 (6, 7). Piezo1/2 ion channels, which sense mechanical changes (15), play an important role in the 5-HT system by regulating the expression of the chemicals at protein and genetic levels. We investigated TPH1, an enzyme involved in the 5-HT synthesis. 5-HT is synthesized from tryptophan by TPH (30). TPH1 is an important isoform in ECs. Our study showed that the expression of TPH1 was elevated in ECs after the addition of GsMTx4. This may directly affect the synthesis of 5-HT. We examined the signaling pathways that might be involved in this process. GPCRs are activated to induce 5-HT secretion (31). Adenosine (32), purinergic signaling (33) and uridine triphosphate (34) can also play a regulatory role in the synthesis and secretion of 5-HT. As a permeable ion channel, the influx of calcium ions is a feature of Piezo1/2 activation. Many calcium-related signaling pathways (35) regulate gene transcription, excitability, cytokinesis, apoptosis, and motility. Four molecules associated with calcium signaling were studied at the mRNA level: AMPK, IP3R, MAPK, and PKC (protein kinase C). We observed a significant increase in the expression of MAPK. Furthermore, we investigated the phosphorylation levels of important molecules involved in the three MAPK pathways. Among them, the phosphorylation levels of p38 were elevated, whereas the phosphorylation levels of JNK and ERK were not significantly different. This suggests that the p38 signaling pathway is involved in the physiological process of 5-HT elevation. In future studies, a more extensive examination of the MAPK signaling pathway could deepen our understanding of the mechanisms underlying the regulation of 5-HT levels by Piezo1/2. Our experiments focused on the long-term inhibition of the Piezo ion channel and its impact on 5-HT in QGP-1 cells. When we observed an elevation in 5-HT levels, it contradicted the effects expected from the transient activation of Piezo1 and Piezo2. We speculated that this discrepancy might be related to other compensatory mechanisms. In the experimental design, we examined the expression levels of the Piezo ion channel and the TPH1, revealing the influence of GsMTx4 on protein expression. Additionally, we further validated our hypothesis by assessing the levels of relevant signaling pathway molecules. Clearly, this result is intriguing, as the long-term inhibition of the Piezo ion channel in QGP-1 cells led to an increase in 5-HT levels. Our experiments can only explain a portion of the mechanism, and a deeper understanding requires further experimental design and validation.

Finally, GsMTx4 altered intestinal dynamics *in vivo*. Our study found that the addition of GsMTx4 had no effect on the gross morphology of the intestine or intestinal inflammation, nor did it alter the nature of the stool in terms of number or quality under *in vivo* conditions. However, the intestinal passage time was significantly prolonged. This showed that GsMTx4, which inhibits the function of the Piezo1/2 ion channel, also prolonged intestinal passage time. This effect can be attributed to two aspects. The first condition involves the amount of 5-HT. 5-HT directly influences the dynamics of the intestine (36–39) by increasing intestinal propulsion and segmental movement. By increasing the amount of 5-HT, the intestinal motility can be directly altered. However, enterochromaffin cells are relatively scarce in the intestine, and our experiments *in vitro* did not result in a significant increase in 5-HT. The physiological effects of such a low-level increase in 5-HT need further validation. The second condition involved the direct effect of Piezo2 on intestinal motility. Inhibition of Piezo2 expression in epithelial cells can attenuate intestinal motility (16). GsMTx4 may inhibit other components of the Piezo ion channel in the intestine, such as neurons, which play a crucial role in regulating gastrointestinal motility. Our experiments did not precisely inhibit the Piezo ion channel in enterochromaffin cells, which could lead to different experimental outcomes.

In future experiments, we hope researchers can make improvements in two aspects. Firstly, by employing transgenic technology, they can precisely study the function of enterochromaffin cells and investigate the physiological effects resulting from the knockout of the Piezo ion channel. Secondly, we encourage more in-depth investigations into the relationship between enterochromaffin cells and the Piezo ion channel using animal models or human samples.

## Data availability statement

The raw data supporting the conclusions of this article will be made available by the authors, without undue reservation.

## Ethics statement

The animal study was approved by Institutional Animal Care and Use Committee of Zhejiang University. The study was conducted in accordance with the local legislation and institutional requirements.

## Author contributions

ZZ and MJ contributed to conception and design of the study. ZZ, XC, CH, RG and YW performed the experiments. ZZ, XC, ZL and XS performed the statistical analysis. ZZ wrote the first draft of the manuscript. XC, SC, CH, RG and MJ wrote sections of the manuscript. All authors contributed to the article and approved the submitted version.
